# Stretchable and Self-Healable Graphene–Polymer Conductive Composite for Wearable EMG Sensor

**DOI:** 10.3390/polym14183766

**Published:** 2022-09-08

**Authors:** Jihyang Song, Yewon Kim, Kyumin Kang, Sangkyu Lee, Mikyung Shin, Donghee Son

**Affiliations:** 1 Department of Superintelligence Engineering, Sungkyunkwan University (SKKU), Suwon 16419, Korea; 2 Center for Neuroscience Imaging Research, Institute for Basic Science (IBS), Suwon 16419, Korea; 3 Department of Electrical and Computer Engineering, Sungkyunkwan University, Suwon 16419, Korea; 4 Department of Intelligent Precision Healthcare Convergence, Sungkyunkwan University, Suwon 16419, Korea; 5 Department of Biomedical Engineering, Sungkyunkwan University, Suwon 16419, Korea

**Keywords:** composite, graphene, self-healing, stretchability, electromyogram, human–robot interface

## Abstract

In bioelectronics, stretchable and self-healable electrodes can reliably measure electrophysiological signals from the human body because they have good modulus matching with tissue and high durability. In particular, the polymer–graphene composite has advantages when it is used as an electrode for bioelectronic sensor devices. However, it has previously been reported that external stimuli such as heat or light are required for the self-healing process of polymer/graphene composites. In this study, we optimized a conducting composite by mixing a self-healing polymer (SHP) and graphene. The composite materials can not only self-heal without external stimulation but also have rapid electrical recovery from repeated mechanical damage such as scratches. In addition, they had stable electrical endurance even when the cyclic test was performed over 200 cycles at 50% strain, so they can be useful for a bioelectronic sensor device with high durability. Finally, we measured the electromyogram signals caused by the movement of arm muscles using our composite, and the measured data were transmitted to a microcontroller to successfully control the movement of the robot’s hand.

## 1. Introduction

Bioelectronic healthcare devices [1,2,3,4,5,6] such as biosensors [7,8,9,10,11,12,13,14,15], bio-implants [16,17], and human–machine interface systems [18,19] that can measure human electrophysiological signals for health monitoring and feedback have gained attention in recent years. Compared to rigid metal-based electronic devices, flexible and stretchable bioelectronic devices are soft and thin, so they are comfortable to wear and can improve treatment efficiency [20,21,22,23]. For this reason, although various groups have reported flexible and stretchable conductors using different approaches [23,24,25,26,27], they are still susceptible to mechanical damage such as scratches or cutting. Therefore, in addition to intrinsic stretchability and conductivity, self-healing properties are required to restore damaged functions and extend lifespan, so new bioelectronic devices based on self-healing materials have emerged [28,29,30]. Composite materials can allow these devices to have unique properties [31,32,33,34]. For instance, composite materials with conductivity, softness, and stretchability were easily formed by mixing an insulating polymer matrix and conductive fillers such as metal powders or carbon materials [35,36,37,38,39]. In particular, a graphene-based composite has gained much attention for use in bioelectronic devices [40,41,42].

Graphene is a two-dimensional (2D) carbon nanomaterial with a honeycomb structure bonded by hybridized sp^2^ orbitals of C=C double bonds, and it has unique electrical/structural characteristics such as a high Young’s modulus, mechanical strength, excellent electrical conductivity, flexibility, transparency, and biocompatibility [43,44,45]. Due to these characteristics, graphene has attracted considerable attention as a conductive filler for polymer composites [46,47], especially in soft bioelectronics. Moreover, several results have been reported about self-healable polymer–graphene composites [48,49,50,51,52]. However, most of these depend on external energy sources or stimulation such as microcapsules, light (UV, microwave, etc.), heat, and so on. For example, Zhang et al. reported a functionalized graphene–polyurethane (PU) composite that can self-heal by a photothermal reaction using an infrared (IR) laser source [50]. However, its healing process needs very high-temperature conditions (150 °C) from the focused laser source to facilitate Diels–Alder chemistry. Valentini et al. showed self-healable silicone rubber–graphene composites, but they also need a high-temperature oven that can raise the temperature up to 250 °C for 2 h [51]. Pan et al. demonstrated that graphene oxide-based polyacrylamide hydrogels could be self-healed at room temperature, but their healing efficiency significantly dropped (approximately from 92% to 45.6%) without water moisture conditions [52].

In this study, we present a self-healable, stretchable, and conductive nanocomposite for an electromyogram (EMG) sensor by the drop-casting of a tough self-healing polymer (PDMS-MPU_0.4_-IU_0.6_, SHP) [53]–graphene composite solution. The optimized composite has low initial resistance (~40.5 Ω) and can be stretched to harsh tensile strain (~100%). Owing to the low glass transition temperature of the SHP matrix [35,36], our SHP–graphene composite also showed its autonomous self-boosting during mechanical deformation (~50% strain) by the recovery of percolative electrical pathways in dynamic polymer networks. This self-healing process did not need any external sources and was enabled at room temperature. Additionally, our composite has stable electrical performance in cyclic stretching tests after self-healing without the introduction of a bilayer structure. Finally, we successfully demonstrated that the EMG signal can be monitored by coating the surface of the SHP–graphene composite with alginate hydrogel. The signal was used by the robot to mimic human hand motions.

## 2. Materials and Methods

### 2.1. Preparation of the Conductive SHP–Graphene Composite

All chemicals and solvents were purchased from Sigma-Aldrich (Burlington, MA, USA). Graphene nanopowder (Grade A0–4, GRAPHENE SUPERMARKET, Ronkonkoma, NY, USA) was used as a conductive filler on polymer composites. Octadecyltrichlorosilane (OTS)-treated silicon oxide wafer was prepared following previous reports [54]. Firstly, silicon wafer was cleaned with acetone and isopropyl alcohol. Its surface was treated by oxygen plasma (100 W, 200 mTorr, 2 min) using a reactive ion etcher (Scientific engineering, Suwon, Korea) and the wafer was immersed in a mixture solution of OTS/n-hexane (0.5% *v*/*v*) for one hour. After the immersion, the wafer surface was cleaned by ethyl alcohol and gently dried with pure nitrogen gas. Then, the wafer was annealed at 120 °C for 30 min on a commercial hot plate. Finally, it was sonicated in chloroform for 5 min.

**Figure 1:**
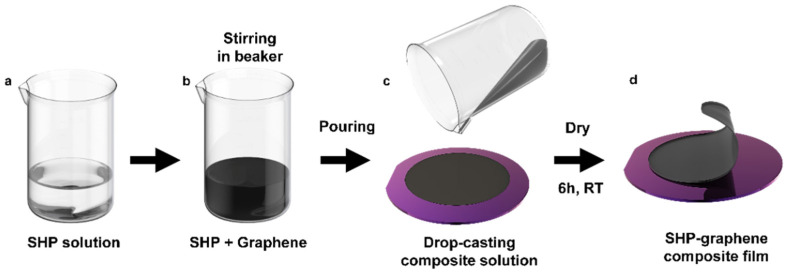
Schematics for the fabrication processes. (**a**) Stirring SHP solution dissolved in chloroform. (**b**) Introduction of graphene powders in the beaker. (**c**) Drop-casting on OTS-treated wafer. (**d**) Peeling off the SHP–graphene composite on the wafer after 6 h.

Following the wafer treatment, the SHP [53] was dissolved in chloroform for 1 h (Figure 1a). After this, the SHP solution and graphene were mixed at the weight ratios of SHP:graphene (1:0.3, 1:0.5, 1:0.7, 1:0.9) and stirred for 4 h (Figure 1b). The solution was then poured onto the OTS-treated wafer to evaporate the solvent at room temperature (Figure 1c) and the completed composite was peeled off from the wafer (Figure 1d).

### 2.2. Electrical and Mechanical Characterizations of the Conductive Composite

All electrical properties of prepared SHP–graphene composite with each ratio were measured using a digital multimeter (Keithley 2450 Digital Multimeter, Clackamas, OR, USA). Stretching test and cyclic durability (0% to 50% strain over 200 cycles) tests to characterize the electrical properties of the samples were performed using a motorized X-conversion stage and corresponding software (Jaeil Optical Corp., Daegu, Korea). The source meter was used to monitor real-time resistance changes and cyclic endurance during the stretching. The initial width and length of the sample were 2 mm and 3 mm, respectively, and the sample was loaded on the stretcher using double-sided tape (3M, Maplewood, MN, USA). The stretching rate of both tests was 60 mm/min.

We measured the tensile stress per strain of our composite to evaluate mechanical properties. The experiments were performed with a universal tensile machine (UTM, Instron 34SC-1, Norwood, MA, USA) for continuous stretching at a speed rate of 5 mm/min. The initial length and width of the composite were 30 mm and 10 mm, respectively, and the length after loading the sample on chuck was 5 mm. Optical microscope (OM) (BX51, Olympus, Japan) and Field Emission Scanning Electron Microscopes (FE-SEM) (JSM-IT800, JEOL Ltd., Tokyo, Japan) were used to confirm that the optimized composite can be self-healed even after mechanical damage.

### 2.3. Fabrication of Polymer–Hydrogel Hybrid Sensor

To coat the surface of the composite, 0.5 g of sodium alginate (SA; Sigma-Aldrich, Burlington, MA, USA) was dissolved in 4.5 mL of DI water (10 wt%). After the dissolution of sodium alginate, it was coated on the top surface of our composite.

### 2.4. Measurement and Processing of Human Skin EMG Signals

The alginate-coated SHP–graphene composite was fixed to the skin using Tegaderm film (3M, Maplewood, MN, USA) for EMG measurement. The signals were recorded using a bio-signal amplifier (Bio Amp FE231, AD Instruments, Dunedin, New Zealand) and data acquisition device (PowerLab 8/35, AD Instruments). Action potential signals were filtered on the authority of the ISEK (International Society of Electrophysiology and Kinesiology) standard (1500-Hz low-pass filter). We used LabChart 8 Pro (AD Instruments, Bella Vista, New South Wales, Australia) software to obtain all data. The obtained data were transferred to a conventional microcontroller (Arduino Mega 2560) that moves the robot’s hand (DFRobut, Shanghai, China). The authors obtained Institutional Review Board (IRB) approval (NO. SKKU 2022-07-035) from Sungkyunkwan University for measuring EMG signals.

### 2.5. Impedance−Frequency Measurement of the Composite

To analyze the electrochemical impedance (EIS) of our composite, a potentiostat (ZIVE SP1, WonATech, Seoul, Korea) was used. For EIS measurement, a commercial Ag/AgCl electrode as a reference, Pt wire as a counter electrode, and our composite as a working electrode were placed in phosphate-buffered saline 1 × (PBS) solution (pH 7.2, Biosesang, Seongnam-si, Gyeonggi-do, Korea). The SHP–graphene composite and alginate-coated composite were prepared as the samples to be analyzed. The area of the samples was 1 cm × 1 cm and the frequency range of this EIS measurement was from 0.1 Hz to 10 kHz with an amplitude of 10 mV. In addition, The EIS spectroscopy with an equivalent circuit model was performed to confirm the change in the impedance spectrum depending on the strain. The Randles equivalent circuit model could be adapted for modeling the impedance transformation of the composite. We observed the impedance spectra of the composite after the applied strain was maintained for 10 min. The composite was gradually stretched to 20% strain each time.

## 3. Results and Discussion

### 3.1. Electrical and Mechanical Characteristics of SHP–Graphene Composite to Optimize Weight Ratio

**Figure 2:**
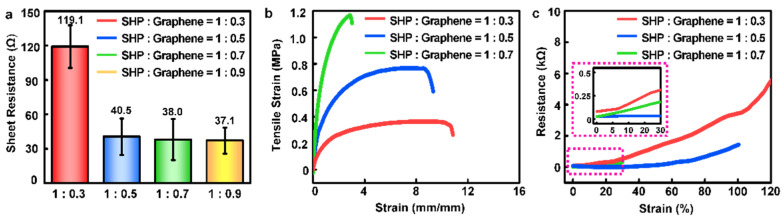
Electrical and mechanical characterizations of a stretchable conductive composite. (**a**) Sheet resistance, (**b**) stress–strain curve, and (**c**) changes in strain-dependent resistance of SHP–graphene composites with different weight ratios of graphene.

Figure 2 shows the optimized process of the SHP–graphene composite. The initial sheet resistance (Figure 2a) was measured to investigate the saturation point of the electrical characteristics on the composite according to the weight ratio. When the weight ratio of graphene in the composite exceeded a certain level, the maintenance of the initial resistance was discovered regardless of the weight ratio. When the low graphene contents were applied to the composite (SHP:graphene = 1:0.3), high resistance was shown (119.1 Ω). However, the composites with higher graphene contents (SHP:graphene = 1:0.5 and SHP:graphene = 1:0.7) had similar resistance values. This suggests that the conductivity of the composite was saturated at a certain ratio of graphene. Next, the mechanical properties were confirmed by stretching the composites with various weight ratios (from 1:0.3 to 1:0.7) at a speed of 5 mm/min (Figure 2b). When the strain was applied, the SHP graphene = 1:0.5 showed better stretchability (~100% strain) than SHP:graphene = 1:0.7 (~30% strain). It is noted that the former composite also showed a lower modulus (softness) than that of the latter one. Thus, we hypothesized that the SHP:graphene = 1:0.5 is best to use as an electrophysiology sensor on human skin. Not only that but it also maintained electrical properties with 100% strain (Figure 2c). In summary, the maximum elongation length of the sample decreased, and electrical conductivity was improved (resistance was sharply decreased) as the relative amount of graphene increased in the composite. There was also an optimum saturation point of the electrical characteristics on our composite. Therefore, the composite with a medium ratio (SHP:graphene = 1:0.5) was adopted to measure EMG signals because of its saturated initial resistance (~40.5 Ω) and lower graphene ratio compared with other saturated ratios.

### 3.2. Self-Healability of the Optimized Conductive Composite

**Figure 3:**
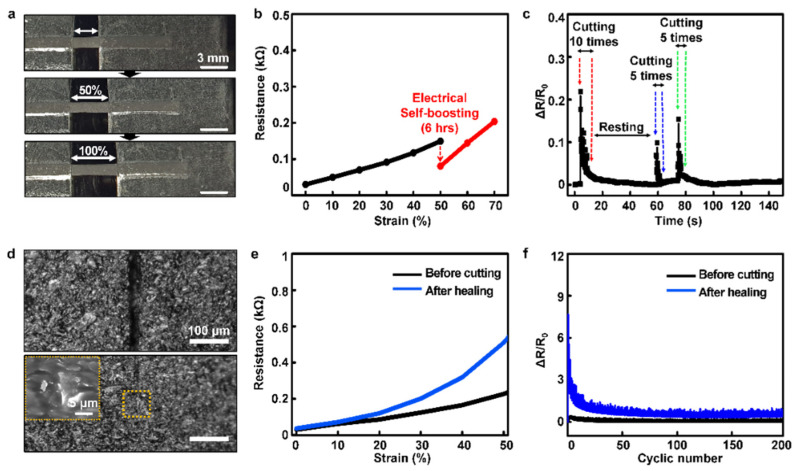
Characteristics of optimized conductive composites: (**a**) Photographs of optimized SHP–graphene composite conductor stretched up to 100% strain. (**b**) Resistance–strain data of the composite showing electrical self-boosting properties at 50% strain. (**c**) Electrical recovery phenomenon of the composite after several times of cutting. (**d**) OM images of SHP–graphene composite before and after self-healing (24 h). (Inset: SEM image of the composite after self-healing.) (**e**) Resistance–strain graphs of the composites before cutting and after self-healing. (**f**) Repetitive stretching (strain of 50%, over 200 cycles) and releasing test of the composites before cutting and after self-healing.

Figure 3 describes the self-healing characteristics of our optimized composite. We confirmed that the composite showed uniform electrical resistance values on every position (Supplementary Figure S1). The optimized SHP–graphene composite maintained its electrical performance (1.44 kΩ) despite the harsh strain (~100%) (Figure 2c, blue line and Figure 3a). In previous reports, it was demonstrated that the low crosslink density of the polymer matrix allows for dynamic movement of the graphene nanosheets as a response to deformation in a time-dependent manner [40]. In addition, it was reported that when the nanocomposite conductor is stretched, the conductive fillers are rearranged in the SHP matrix over time due to stress relaxation in the dynamically crosslinked polymer matrix [35]. Through this rearrangement, self-recovery of conductivity under tensile strain, which is called electrical self-boosting phenomenon, occurred. Similarly, we tested the electrical self-boosting phenomenon under a stretched condition when graphene rearranges and electrical recovery occurs in the SHP matrix (Figure 3b and Supplementary Figure S2). We observed that the resistance drops from 150 Ω to 80 Ω at 50% strain. Moreover, the composite has a rapid recovery of electrical properties even when scratches are repeatedly applied (Figure 3c). These results show that the SHP–graphene composite not only has conductivity and stretchability but also has a particular characteristic of recovering electrical property to a certain extent despite mechanical damage. Next, we cut the surface of our composite with a razor blade to evaluate the mechanical and electrical self-healing properties of the optimized composite. Dissected samples of two pieces (Figure 3d, top) verified that the graphene network was reconstructed (Figure 3d, bottom) after 24 h at room temperature by OM and SEM images. Figure 3e shows that both mechanical and electrical recovery were achieved. Even after mechanical damage, the initial resistance was similar and the change in resistance due to deformation was not significantly different. Additionally, the composite exhibited high initial ΔR/R_0_ values under harsh conditions but exhibited excellent durability over time (Supplementary Figure S3). More surprisingly, the self-healable composite conductor had a stable electrical performance both as new and after self-healing for 200 cycles at 50% strain (Figure 3f). We were able to confirm that the optimized SHP–graphene composite recovered not only mechanical properties but also electrical properties after damage through self-healing.

### 3.3. Robust Interactive Human–Robot Interface Based on Stretchable and Self-Healable Conductive Composite

**Figure 4:**
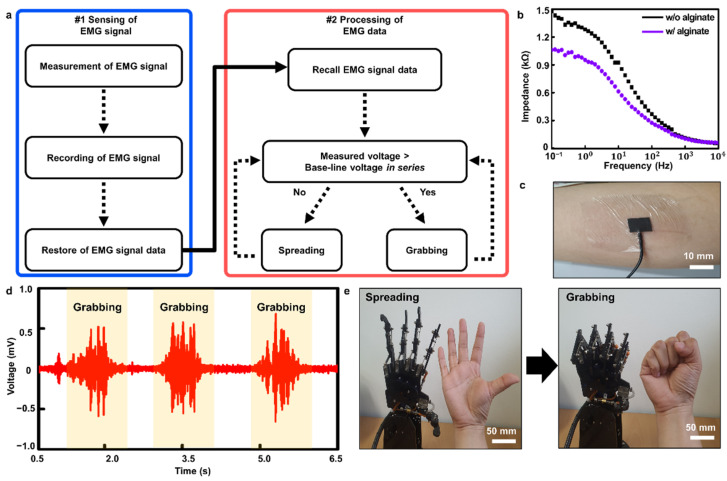
Demonstration of human−robot interface with stretchable and self-healable electrodes. (**a**) Flow diagram of EMG signal recording and processing. (**b**) Impedance data of the composite with and without alginate. (**c**) A photograph of the electrode attached on the skin. (**d**) Recorded EMG signals using the electrode. (**e**) Demonstration of the human−robot interface (spreading and grabbing).

Through biometric monitoring of the human body, medical information related to the patient’s health condition can be obtained [55]. In particular, EMG signals are becoming increasingly important in wearable bioelectronics as they are useful in many applications, including prosthetic or rehabilitation devices, and human–machine interfaces [56,57,58,59]. In particular, in this area, stretchable and self-healable electrodes are practical because they can enhance the durability and quality of the device without discomfort while wearing [35]. To match these advantages, our SHP–graphene composite also showed excellent electrical durability under harsh conditions as mentioned above. Additionally, we measured impedance to find the electrochemical properties of our composite to be used when recording the EMG signals (Supplementary Figure S4). In addition, Randles equivalent circuit was modeled using the Nyquist plot to analyze our electrode characteristics (Supplementary Figure S5). Although the impedance value slightly increased as the strain was applied, it maintained a stable degree without an electrical breakdown. The modeled Randall equivalent circuit also showed a similar trend in the bulk resistance (R_b_), electric double layer capacitance (C_dl_), and charge transfer resistance (R_ct_) values [60]. Through these characteristics, it can be seen that our composite is appropriate to be used as an EMG electrode. Figure 4a depicts a flowchart of our system.

Incidentally, alginate hydrogel is widely employed for biomedical sensors because of its ionic conducting property and excellent biocompatibility [61,62,63]. We coated alginate hydrogel on the surface of our composite to reduce the electrochemical impedances of the electrode when attached to the skin. To make alginate solution, sodium alginate was dissolved in DI water to make alginate hydrogel with a concentration of 10 wt% (Supplementary Figure S6). If the concentration of alginate was lower than 10 wt%, it was too thin, and if it was high, it would agglomerate and could not be coated uniformly on the surface of the composite. Furthermore, we observed that the 10 wt% concentration of the alginate-coated composite had lower impedance than the bare SHP–graphene composite (Figure 4b). In addition, we prepared sensing (SE), reference (RE), and ground electrodes with a size of 1 cm × 1 cm for EMG measurement (Supplementary Figure S7).

An EMG electrode (i.e., SHP–graphene composite with an alginate layer) was attached to the arm to detect the contraction and relaxation of the muscles (Figure 4c). We monitored the EMG signal by performing two designated motions (grabbing and spreading). During hand grabbing, the voltage of the signal was higher than the baseline. On the other hand, the voltage was lower and stable during spreading. This means we obtained EMG signals that were clearly distinguished according to the contraction and relaxation of the muscles (Figure 4d). At this time, signals were collected and filtered using a bio-signal amplifier and a data acquisition device. We also implemented robotic hand motions through the detected EMG signal (Figure 4e). The measured data were transmitted to the microcontroller, and by using these data, the robot’s hand could properly perform. In other words, the filtered data are passed to the microcontroller that moves the robot, causing the robot to mimic human hand motions (grabbing and spreading).

## 4. Conclusions

We prepared SHP−graphene composite electrodes for biomedical EMG sensors that can be attached to human skin to monitor and provide feedback on human health. Our SHP−graphene composite has many advantages for use as a soft bioelectrode: (1) It is easily made by a simple mixing process. (2) It can autonomously self-heal itself at room temperature. (3) It has excellent electrical recoverability even from repeated scratches. (4) It exhibits stable durability even after cutting. (5) It shows a stable value even after stretching. Based on these properties, the EMG signal measurement was successfully demonstrated with alginate hydrogel. Moreover, the movements of the robot were realized using EMG signal data from the contraction of muscles of the forearm flexor. Therefore, we believe that our composite is a suitable candidate for soft bioelectronic devices for monitoring physiological signals in the human body.

## Data Availability

The data presented in this study are available on request from the corresponding author.

## Supplementary Materials

The following supporting information can be downloaded at: https://www.mdpi.com/article/10.3390/polym14183766/s1, Figure S1: Uniformity of optimized SHP–graphene composite conductor; Figure S2: Resistance-strain data of the composite held for 20 min at 20% intervals; Figure S3: Cyclic durability of the optimized composite; Figure S4: Impedance−frequency data of the composite according to the strain; Figure S5: Characterization of the electrode according to the strain; Figure S6: Photographs of alginate solution according to concentration; Figure S7: Schematic of electrode positioning for EMG monitoring.
