# Supplementary material for: Stretchable and Self-Healable Graphene–Polymer Conductive Composite for Wearable EMG Sensor
Source: Polymers (Basel). 2022 Sep 8;14(18):3766. doi: 10.3390/polym14183766 (PMC9505217; doi:10.3390/polym14183766)
Supplement: Supplementary file 1 [file polymers-14-03766-s001.zip › polymers-1871436-supplementary.pdf]

Supplementary Materials

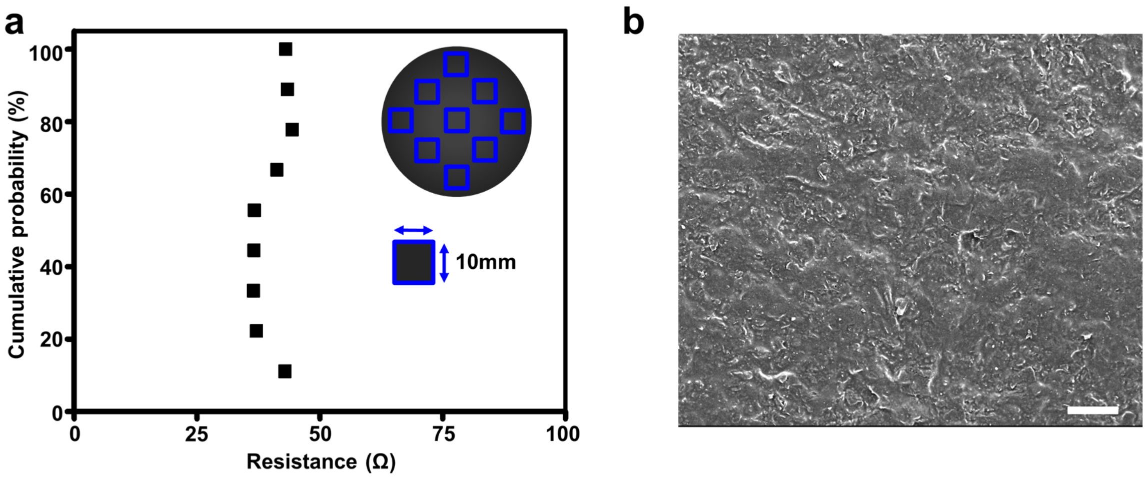

Figure S1. Uniformity of the optimized SHP–graphene composite conductor. (**a**) Resistance measurement of 9 different points on the conductor film for area uniformity evaluation. (**b**) SEM image of the SHP–graphene composite surface (Scale bar: 100 μm).

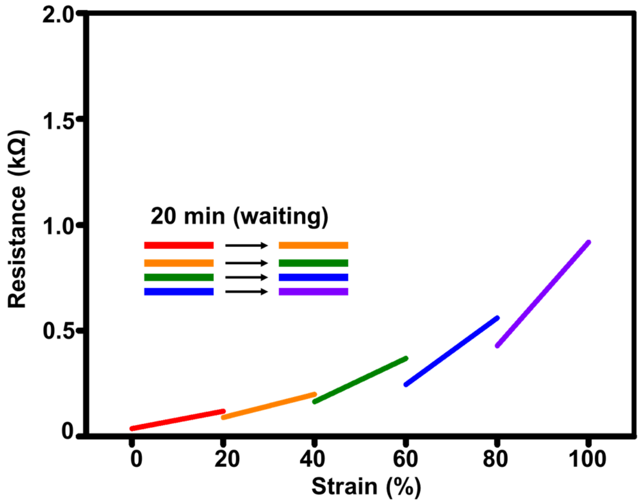

Figure S2. Resistance–strain data of the held composite for 20 min at 20% intervals. After stretching at 20% intervals, the resistance of the composite gradually decreased during holding for 20 min.

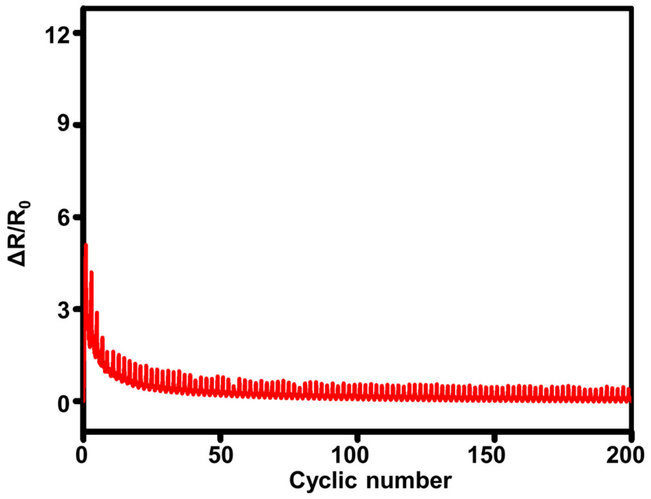

Figure S3. Cyclic durability of the optimized composite. To verify that the electrical and mechanical durability is stable even under repeated 0% to 100% strain.

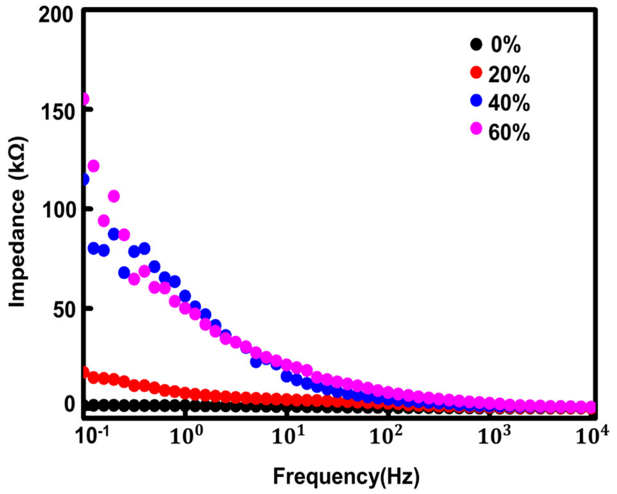

Figure S4. Impedance–frequency data of the composite according to the strain. Impedance measured according to the frequency of the optimized composite at strain of 0% (black), 20% (red), 40% (blue), 60% (pink).

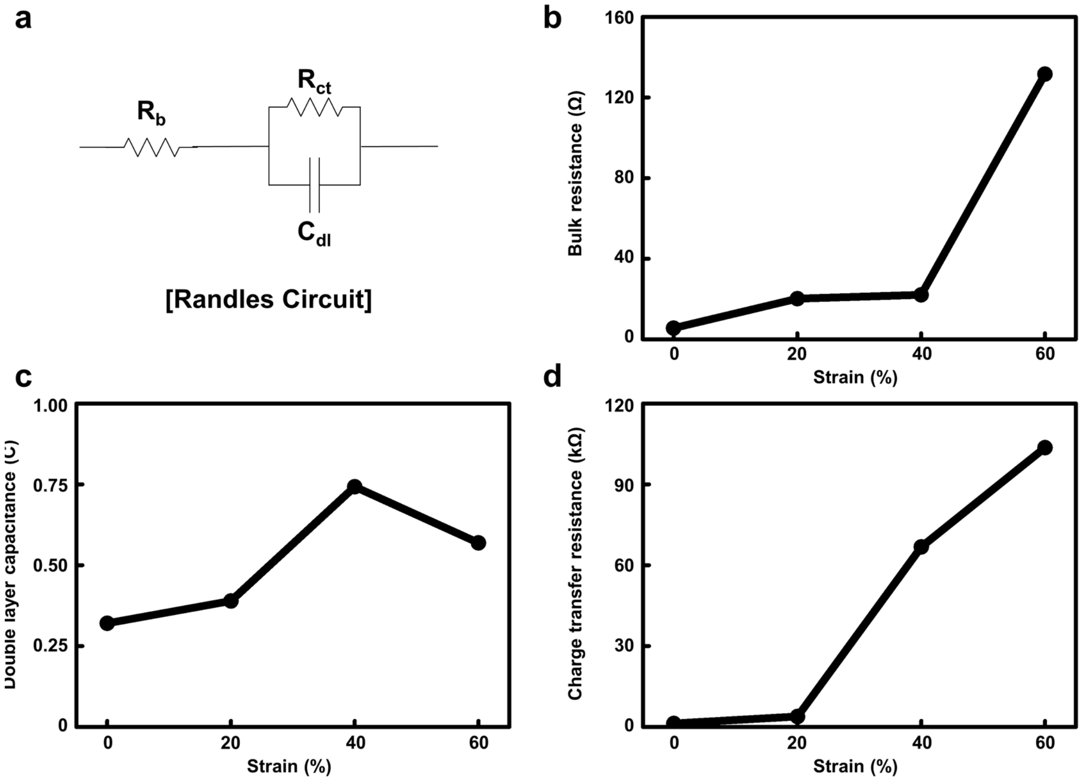

Figure S5. Characterization of the electrode according to the strain. (**a**) The equivalent circuit model consisting of bulk resistance (R_b), electric double layer capacitance (C_{dl}) and charge transfer resistance (R_{ct}). (**b**) Bulk resistance value according to the strain. (**c**) Double layer capacitance value according to the strain. (**d**) Charge transfer resistance value according to the strain.

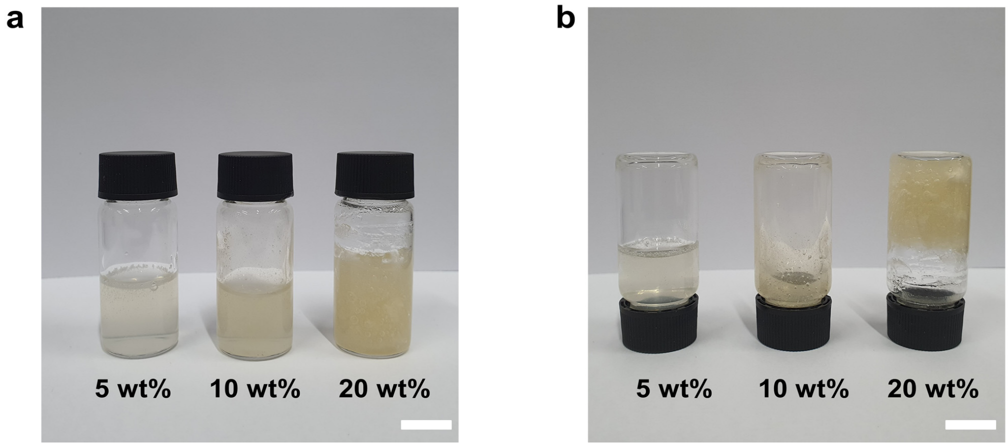

Figure S6. Photographs of the alginate solution according to concentration. (**a**) 5 wt% (left), 10 wt% (middle) and 20 wt% (right) of the alginate solution, respectively. (**b**) Right after turning over vials (Scale bar: 1 cm).

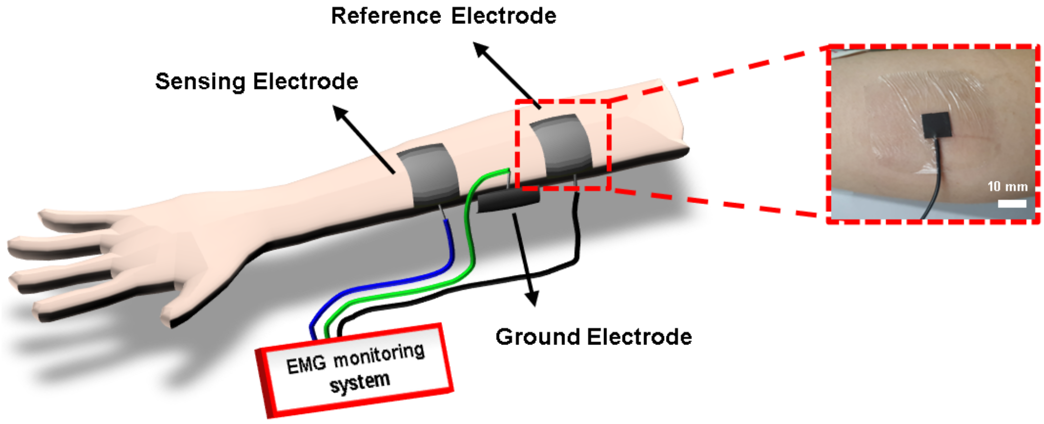

Figure S7. Schematic of electrode positioning for EMG monitoring. Illustration of positioning the three-electrode (sensing, reference, and ground) and a photograph of the composite electrode. (Scale bar: 10 mm).
